# Pulse Crop Effects on Gut Microbial Populations, Intestinal Function, and Adiposity in a Mouse Model of Diet-Induced Obesity

**DOI:** 10.3390/nu12030593

**Published:** 2020-02-25

**Authors:** John N. McGinley, Vanessa K. Fitzgerald, Elizabeth S. Neil, Heather M. Omerigic, Adam L. Heuberger, Tiffany L. Weir, Rebecca McGee, George Vandemark, Henry J. Thompson

**Affiliations:** 1 Cancer Prevention Laboratory, Colorado State University, Fort Collins, CO 80523, USA; john.mcginley@colostate.edu (J.N.M.); vanessa.fitzgerald@colostate.edu (V.K.F.); elizabeth.neil@colostate.edu (E.S.N.); 2 Department of Horticulture, Colorado State University, Fort Collins, CO 80523, USA; heather.omerigic@colostate.edu (H.M.O.); adam.heuberger@colostate.edu (A.L.H.); 3 Department of Food Science and Human Nutrition, Colorado State University, Fort Collins, CO 80523, USA; tiffany.weir@colostate.edu; 4 USDA-ARS Grain Legume Genetics and Physiology, Washington State University, Pullman, WA 99164, USA; rjmcgee@wsu.edu (R.M.); george.vandemark@ars.usda.gov (G.V.)

**Keywords:** adiposity, *Akkermansia muciniphila*, Bacteriodetes, chickpea, common bean, dry pea, Firmicutes, intestinal function, lentil, pulses

## Abstract

The dietary fiber gap that is present in many countries co-exists with a low intake of grain legumes (pulses) that have 2–3 times more dietary fiber than cereal grains that are commonly recommended to increase fiber intake. Given the relationships among dietary fiber, gut health and chronic disease risk, a study was undertaken in a preclinical mouse model for obesity to examine how commonly consumed pulses, i.e., chickpea, common bean, dry pea and lentil, would impact gut microbes, intestinal function, and adiposity. Pulses were fed to C57BL/6 mice at similar levels of protein and fiber. Bacterial count in the cecum was elevated 3-fold by pulse consumption. At the phylum level, a 2.2- to 5-fold increase in Bacteriodetes relative to Firmicutes was observed. For *Akkermansia muciniphila*, a health-beneficial bacterium, differential effects were detected among pulses ranging from no effect to a 49-fold increase. Significant differences among pulses in biomarkers of intestinal function were not observed. Pulses reduced accumulation of lipid in adipose tissue with a greater reduction in the subcutaneous versus visceral depots. Metabolomics analysis indicated that 108 metabolites were highly different among pulse types, and several compounds are hypothesized to influence the microbiome. These results support recent recommendations to increase consumption of pulse-based foods for improved health, although all pulses were not equal in their effects.

## 1. Introduction

In many regions of the world, grain legumes, also referred to as pulses, are neglected staple foods that have the potential to directly impact food insecurity while helping individuals achieve healthy and balanced diets and to accelerate progress towards the Sustainable Development Goals of the WHO/FAO [1,2,3,4,5]. While pulses are frequently promoted for their content of protein and micronutrients, they are also an excellent source of dietary fiber, a fact that is under appreciated [6]. Since the seminal work of Burkitt and colleagues, there has been an awareness of the role of dietary fiber in maintaining the physiological function of the mammalian intestine [7]. However, recognition of the importance of dietary fiber for human health and well-being has never risen to the establishment of fiber as an essential nutrient. Nonetheless, a recommended level of dietary fiber has been proposed (14 g/1000 kcal) and many systematic reviews and meta-analyses support the health benefits associated with cohorts that meet or exceed this recommendation [8,9,10,11,12,13]. Unfortunately, the majority of individuals in developed countries such as the United States fail to meet the recommended level of intake for dietary fiber and the magnitude of the gap is large, an approximately 50–70% shortfall [6]. This gap has been resistant to change, despite decades of public health interventions and development of fiber enriched food products. Recently, we have advocated more focus on pulse crops to not only close the dietary fiber gap, but to extend intake above recommended levels to those that have been reported to significantly reduced chronic disease mortality [14]. However, to achieve such intakes, i.e., 50–80 g total dietary fiber per day, we argue that a better understanding of the effects of high fiber foods such as pulses is needed. We recently published a comparative analysis of the dietary fiber content of chickpea, common bean, dry pea, and lentil, and reported that differences existed among pulses that may be of importance to various sectors of the food systems industry [6,15]; however, from the consumers’ perspective, none of the differences observed negate the conclusion that pulses are an abundant source of dietary fiber. 

Of the many functions that dietary fiber may affect in mammalian species, there is considerable interest in its influence on the meta-organism, i.e., the gut-associated microbiome [16,17,18,19,20]. The available literature on this topic is expanding rapidly, e.g., [21,22,23,24]; however, we found no studies in which the effects of the four most predominant pulses have been compared. This is important for several reasons. First, there is no consensus on what component(s) of dietary fiber are essential for gut health (reviewed in [6]), and consequently, various reports of dietary fiber composition [21,25,26,27,28] cannot be used to predict gut health effects. Second, a considerable body of literature indicates that gut health effects are attributable to microorganisms that are obligate anaerobes. Thus, except for one report in which microbial analyses were performed on cecal content and stool [21], analyses of the microbiome have been limited to fecal material which may not be directly representative of the profile of microorganisms that colonize the intestinal tract of pulse fed animals/humans and that are responsible for effects of the microbiome on health status. Third, it is widely recognized that results of microbiome analyses can vary widely among publications for a host of technical reasons unrelated to the underlying biology and biochemistry being investigated [29,30,31]; this makes direct comparisons among pulses within the same study of considerable value. Finally, while investigation of cultivars of various pulses is very important, it is equally important to also move the field in a translational direction, i.e., the analysis of each pulse crop as a collection of cultivars that are agronomically important and as might be eaten by the consumer, an approach that agricultural economists refer to as a market basket. In view of these gaps in research, the study reported herein was designed to address three questions when pulses were fed at a similar level of dietary protein and fiber. Do the four pulses: (1) exert the same effect on specific microbial populations that have been implicated in health promotion/disease prevention, (2) impact intestinal function in the same manner, and (3) affect adipose tissue deposition in a mouse model of diet-induced obesity? 

## 2. Materials and Methods 

### 2.1. Experimental Animals 

NCI C57BL/6NCr male mice (21–28 days of age) were obtained from Charles River Laboratories NCI (Frederick, MD, USA). Upon arrival, the mice were fed a purified high-fat diet. This formulation was developed and first reported to induce obesity in this mouse strain by Ulman et al., an effect reported to be accompanied by dysbiosis [32,33,34,35,36,37]. The formulation, which provides 60% of dietary kcal from fat, is commercially available (Catalogue #D12492, Research Diets, Inc., New Brunswick, NJ, USA) [38]. Mice were housed in solid-bottomed polycarbonate rodent cages (42.55 cm long × 20.32 cm wide × 20.32 cm high) that accommodate up to 10 adult mice per cage as defined in the Guide for Use of Laboratory Animals [39]. The animal bedding used was 7090 Teklad sani-chips from Envigo (Indianapolis, IN, USA ). Mice were maintained on a 12 h light/dark cycle at 27.5 ± 2 °C with 30% relative humidity and given ad libitum access to diet and distilled water. All animal studies were performed in accordance with the Colorado State University Institutional Animal Care and Use Committee (protocol 18-7746A).

### 2.2. Experimental Design

As described above, mice were fed the Ulman diet from receipt to 5 weeks of age in order to promote dysbiosis that is associated with the obesity phenotype which manifests beginning 8 weeks of age [40]. At 5 weeks of age, mice were randomized to treatment groups. Mice were either continued on the high-fat (HF) formulation (control diet) or were fed that diet formulation to which common bean, chickpea, dry pea, or lentil was added. The diet formulation has been previously reported [41] and therefore the formulation is detailed in Supplementary Table S1. The formulation of the experimental diets and the rationale for the concentration of pulses has been published [42,43]. Briefly, the level of pulse was (40% w/w), which is approximately twice as high as median intakes of pulse consuming subgroups in the US and Canada. However, the dietary level is actually similar to dietary amounts consumed in developing countries where pulses are dietary staples [43]. Key aspects of the dietary approach were: (1) the inclusion of a low-fat, negative control group, which varied markedly in composition from the high-fat control but that is a recognized control in the B6 DIO model in that it does not induce dysbiosis and has lower obesogenic activity [44], (2) the use of a single common bean market class as a “gold standard” against which to compare the activity of other pulses since we have reported that this common bean market class has anti-cancer and anti-obesogenic activity and that it induces favorable changes in gut microbial populations in this model system [41,45,46], and (3) the creation and investigation of chickpea, dry pea, and lentil using a market basket approach of cultivars that are economically important, but whose identity is unknown by the consumer; thus this reflects what the consumer is likely to experience. Diets were formulated to be similar in protein using proximate analysis data available to the consumer (Supplementary Table S2). Diets containing pulses were supplemented with both the sulfur-containing amino acid, methionine, and with tryptophan, which are respectively the first and second most limiting amino acids in pulse crops [47].

Mice were rehoused at randomization in a single cage per group. Because of aggressive behavior among a few mice, aggressors were removed to prevent injury to the other mice thus resulting in 6–8 mice per group. Mice were fed their respective diets ad libitum throughout the experiment. Body weight data were collected weekly. The experiment was terminated at 22 weeks of age when animals had consumed experimental diets for 17 weeks. 

### 2.3. Necropsy

All the animals in each group were subjected to the following procedures. Mice were not fasted prior to necropsy. Mice were anesthetized using isoflurane inhalation and subsequently euthanized by cervical dislocation. The gastrointestinal tract including the stomach to the superior portion of the rectum was removed. The small intestine was cut at the ileocecal junction and weighed with the stomach attached. A 6.5 cm length of ileum superior to the cecum was excised. A 1 cm section of flushed ileum immediately superior to the cecum was cut and placed in 10% neutral buffered formalin. The remaining ileum was laid on a Kimwipe and a midline incision was made down the entire long axis using blunt tipped scissors. The ileum was reflected, and the edges of the serosa gently tacked down to the Kimwipe. While holding one end of the ileum securely with Adson forceps, a polished stainless-steel spatula was used to gently scrape the ileum mucosa in one continuous motion; contents were placed into a cryovial and snap-frozen in liquid nitrogen for subsequent RNA analysis. The cecum was cut from the colon, weighed and snap-frozen in liquid nitrogen without removal of content in order to retain anaerobiosis with the cecal content. The colon was rinsed in sterile saline and fecal pellets extruded while maintaining proper anatomic orientation. 1 cm sections of colon representing the ascending, transverse and descending portions was excised and fixed in 10% neutral buffered formalin. Inguinal subcutaneous and abdominal visceral adipose tissue were harvested and weighed. In addition, a small portion of these adipose depots were excised and fixed in 10% neutral buffered formalin. Remaining portions of fat depots were snap-frozen in liquid nitrogen. All snap-frozen tissues were stored at −70 °C. The left tibia of each animal was removed, cleaned and measured in mm using a pair of digital calipers in order to normalize the tissue weights. All formalin fixed tissues were processed for histological evaluation.

### 2.4. RNA Transcript Expression 

RNA was isolated from frozen mucosal scrape of the ileum using the RNeasy Mini kit (Qiagen, Germantown, MD, USA) according to the manufacturer’s protocol. Isolated RNA purity (260/280 and 260/230 ratios) and concentration was checked via NanoDrop (Thermo Fisher Scientific, Waltham, MA, USA). RNA integrity was determined using a Bio-Rad Experion (Bio-Rad, Hercules, CA, USA). cDNA was synthesized from 1 µL of total RNA using Superscript II reverse transcriptase (Invitrogen, Carlsbad, CA, USA). DNA oligo primers were synthesized (Integrated DNA Technologies, Coralville, IA, USA), sequences are shown in Supplementary Table S3 [48]. Quantitative real-time PCR was performed on an iCycler (Bio-Rad, Hercules, CA, USA) with optical-grade 96-well plates (Thermo Fisher Scientific, Waltham, MA, USA). Each 20 µL reaction mixture was composed of 5 µL of water, 10 µL 2X SYBR green Supermix (Bio-Rad, Hercules, CA, USA), 1.5 µL each of forward and reverse primers at a final concentration of 0.3 µM, and 2 µL of synthesized cDNA. The following PCR conditions were used: 95 °C for 3 min, followed by 40 cycles of 95 °C for 15 s and 56.5 °C for 1 min. Fluorescent products were detected at the last step of each cycle. qPCR was followed by a melting curve analysis to verify primer specificity under the following conditions: 80 cycles starting at 57 °C for 10 s and increasing by 0.5 °C each cycle. Each sample was run in triplicate and the relative farnesoid X receptor (FXR) expression was calculated by normalizing the threshold cycle (Ct) values to 18S [48]. The fold change in expression relative to the control-fed animals was computed by the delta, delta Ct method as described in [49].

### 2.5. Bacterial Quantification by qPCR

An established approach was used to assess levels of specific bacterial populations. DNA oligo primers coding for 16sRNA sequences unique to *Akkermansia muciniphila*, and bacteria in the phyla Firmicutes and Bacteroidetes, or common to all bacteria were used for qPCR analysis. The DNA oligomers were synthesized by Integrated DNA Technologies, Coralville, IA, and are shown in Supplementary Table S3 [50,51,52]. 

Frozen ceca were removed from the freezer three at a time and allowed to slightly thaw on the bench top, approximately 5–10 min. Small scissors were used to make an incision in the cecum and the mucosa was reflected. A clean, polished stainless-steel spatula was used to remove approximately 100 mg of cecal contents and placed in a dry bead tube and DNA extracted according to the manufacturer’s protocol, QIAamp PowerFecal DNA kit (Qiagen, Germantown, MD, USA). The isolated DNA was checked for purity (260/280 and 260/230 ratios) and concentration via NanoDrop (Thermo Fisher Scientific, Waltham, MA, USA). 

qPCR was performed on cecal DNA using a modified version of the protocol described in [51]. Briefly, qPCR amplification and detection were performed using an iCycler (Bio-Rad, Hercules, CA, USA) with optical-grade 96-well plates (Thermo Fisher Scientific, Waltham, MA, USA). Each 20 µL reaction mixture was composed of 5 µL of water, 10 µL 2X SYBR green Supermix (Bio-Rad, Hercules, CA, USA), 1.5 µL of forward and reverse primers at a final concentration of 0.3 µM, and 2 µL of template DNA (10 ng/µL). PCR conditions were as follows: 95 °C for 3 min, followed by 40 cycles of 95 °C for 15 s, 60 °C for 30 s, and 72 °C for 30 s, and a final extension at 72 °C for 5 min. Fluorescent products were detected at the last step of each cycle. qPCR was followed by a melting curve analysis to verify primer specificity under the following conditions: 80 cycles starting at 5 5 °C for 10 s and increasing by 0.5 °C each cycle. Each sample was run in triplicate, Ct values were normalized to 16S (926 Fwd., 1062 Rev.), and the fold change in expression relative to the control was computed by the delta, delta Ct method as described in [49].

### 2.6. Histology

#### 2.6.1. Histology and Image Acquisition

The tissue fixed in 10% neutral buffered formalin for 24 hours was then processed through a series of graded ethanols, cleared in toluene, embedded in paraffin and sections were cut at 4 µM. Sections were stained with H&E, alcian blue nuclear fast red (AB) [53], and Ki-67 immunostaining as described previously [54]. Images of stained intestine were captured at 200× and images of adipose tissue at 100× magnification using a Zeiss AxioCamHR (Zeiss, Thornwood, NY, USA) digital camera mounted on a Zeiss Axioskop II microscope. Prior to capturing images, background shading correction was achieved using the Zeiss AxioVision software. Images were captured (1300 × 1030 pixels, 24 bit RGB, 150 DPI) and saved as JPEG files.

#### 2.6.2. Morphometric Analysis

A total of ten crypts in ileum, and ascending, transverse and descending colon were analyzed from each animal across serial sections of three different stains listed in Section 2.6.1. Crypts were chosen from multiple image fields along the length of each segment in order to decrease bias and selection was based on the following criteria: (1) a single layer of epithelial cells visible from the base of the crypt to the top of the of villus; (2) patent crypt lumen; (3) visible crypt lamina propria. Ileum and colon crypt height was measured manually from the base of the crypt to the brush border of the uppermost enterocyte nearest the lumen in H&E-stained images using the caliper function in Image Pro Plus v4.5 (Media Cybernetics, Inc. Rockville, MD). AB-stained images depicting crypt goblet cells and intra-crypt mucin were processed using the built-in alcian blue vector in the color deconvolution plugin for ImageJ v1.52d (NIH, Bethesda, MD), resulting in three separate color channel images, red, green and blue. A macro was written in Image Pro Plus v4.5 (Media Cybernetics, Inc. Rockville, MD) to analyze the AB images. Briefly, the blue channel image was imported along with the original AB image. The blue channel image was converted to 8 bit grayscale (256 shades of gray) and contrast applied resulting in a black and white image. Crypts were circumscribed one at a time on the original AB image as an area of interest (AOI). The AOI was copied from the original AB image, pasted to the corresponding XY coordinates in the black and white image and the segmentation range was set 0–254, i.e. black representing alcian blue stained mucin within the crypt against a white (255) background. The software measured the total sum black area in µM^2^ of the AOI and exported the value automatically via dynamic data exchange to an Excel spreadsheet for analysis. To assess cell proliferation, Ki-67 staining was performed as previously described [54] and analyzed using the immunoratio plugin for ImageJ [55]. 

#### 2.6.3. Metabolite Extraction, Detection, and Data Processing

A total of 100 mg of cooked pulse powder was extracted using a MTBE/methanol/water procedure and detected on liquid chromatography–mass spectrometry (LC–MS, positive and negative electrospray ionization modes) and gas chromatography–mass spectrometry (GC–MS, trimethylsilyl/methoximation derivatizations, liquid injections) as described in [56,57]. The data processing procedure is described in [11,58,59]. Briefly, for each sample, raw data files were converted to .cdf format, and a matrix of molecular features as defined by retention time and mass (m/z) was generated using XCMS software in R for feature detection and alignment. Features were grouped using RAMClustR with TIC normalization. LC–MS data were first annotated by searching against an in-house spectra and retention time database using RAMSearch. RAMClustR was used to call the findMain function from the interpretMSSpectrum function (MS-FINDER program v2.40 [60,61]) to infer the molecular weight of each LC–MS compound for annotation of the mass signals. The complete MS spectrum and a truncated MSE spectrum were written to a .mat format for import to MSFinder. The MSE spectrum was truncated to include only masses with values less than the inferred M plus its isotopes, and the .mat file precursor ion was set to the M+H ion for the findMain inferred M value. These .mat spectra were analyzed to determine the most probable molecular formula and structure. MSFinder was also used to perform a spectral search against the FooDB, HMDB, ChEBI, and Lipidmaps metabolite databases (foodb.ca; hmdb.ca; ebi.ac.uk/chebi; lipidmaps.org). All results were imported into R and a collective annotation was derived with prioritization of MSFinder mssearch > MSFinder structure > MSFinder formula > findMain M. All R work was performed using R version 3.3.1 [6]. GC–MS data were annotated using spectral and retention index matching within AMDIS software using the Golm Metabolome Database [62].

### 2.7. Statistical Analyses 

Body mass and morphometric data were evaluated by ANOVA or the Kruskal–Wallis test depending on data distribution (D’Agostino and Pearson omnibus normality test) and Fisher or Dunn’s post hoc test, respectively. Differences were considered significant at *p* ≤ 0.05. Bacterial, and RNA transcript expression data were evaluated by computing fold change (FC) and the significant of differences tested after log transformation and boot strapping to generate confidence intervals [63]. Data analyses were conducted using Systat version 13.0, SAS version 9.2, and Graph Pad Prism, version 5.2. Principal component analysis and orthogonal projection to latent structures analysis of metabolites were conducted using SIMCA v15 software (Sartorius Stedim Biotech, Umea, Sweden), on unit variance scaled data. *z* for each metabolite was calculated as the mean metabolite abundance value within each pulse type minus the mean abundance values of all pulse types divided by the standard deviation of the abundance value all pulse types.

## 3. Results

### 3.1. Effects of Pulses on Growth

**Figure 1:**
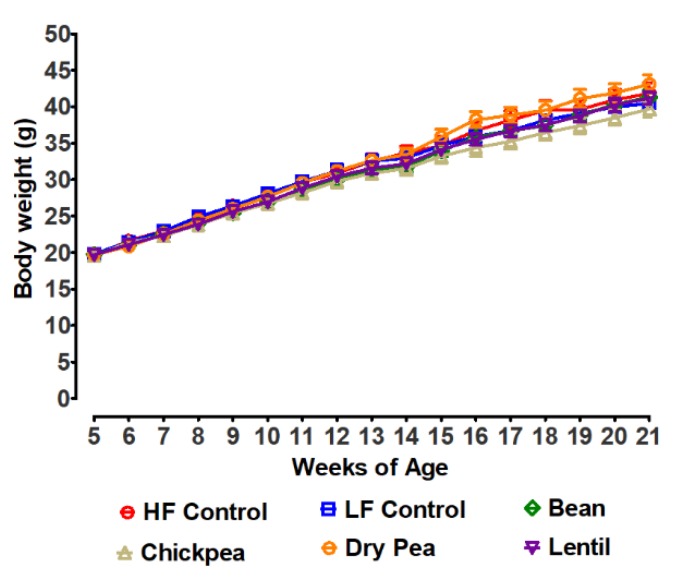
Effect of feeding pulse diets on body weight. HF Control *n* = 7, LF Control *n* = 7, Bean *n* = 6, Chickpea *n* = 7, Dry Pea *n* = 8, Lentil *n* = 8; HF: high fat; LF: low fat. Values are means ± SEM. Differences among groups at each timepoint were not statistically significant.

Mice were assigned to diet groups via the staggered randomization approach using body weight. Thus, group mean body weights were essentially identical when the feeding of experimental diets was initiated at 5 weeks of age. Mice were weighed weekly as shown in Figure 1. At no time point during the study were differences found to be statistically significant among treatment groups.

### 3.2. Effect of Pulses on Cecal Bacteria Populations 

**Figure 2:**
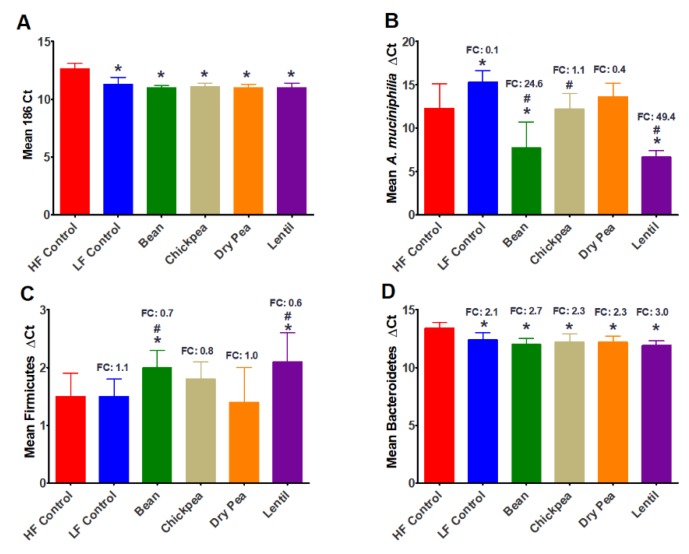
Effect of feeding pulse diets on cecal bacteria populations. (**A**) 16S Ct for 20 ng genomic DNA, mean ± SEM; ANOVA:LF Control *p* < 0.0001, Bean *p* < 0.0001, Chickpea *p* < 0.0001, Dry Pea *p* < 0.0001, Lentil *p* < 0.0001; HF Control vs. all pulses *p* < 0.0001; LF Control vs. all pulses *p* = 0.0443 (**B**) *Akkermansia muciniphilia* ∆Ct, ANOVA: LF Control *p* = 0.0071, Bean *p* = 0.0001, Chickpea *p* = 0.9300, Dry Pea *p* = 0.2131, Lentil *p* < 0.0001; HF Control vs. all pulses *p* = 0.1342; LF Control vs. all pulses *p* = 0.0006 (**C**) Firmicutes ∆Ct, ANOVA: LF Control *p* = 0.7762, Bean *p* = 0.0370, Chickpea *p* = 0.1450, Dry Pea *p* = 0.8696, Lentil *p* < 0.0052; HF Control vs. all pulses *p* = 0.0937; LF Control vs. all pulses *p* = 0.1497 (**D**) Bacteroidetes ∆Ct, ANOVA: LF Control *p* = 0.0003, Bean *p* < 0.0001, Chickpea *p* = 0.0001, Dry Pea *p* < 0.0001, Lentil *p* < 0.0001; HF Control vs. all pulses p = < 0.0001; LF Control vs. all pulses *p* = 0.2189; FC relative to HF control diet; ^*^ different relative to HF control diet (*p* ≤ 0.05); ^#^ different relative to LF control diet (*p* ≤ 0.05); FC calculated relative to HF Control; HF Control *n* = 7, LF Control *n* = 7, Bean *n* = 6, Chickpea *n* = 7, Dry Pea *n* = 8, Lentil *n* = 8; FC: fold change; HF: high fat, LF: low fat.

Pulse consumption induced a 3-fold increase in the bacterial content of the cecum relative to the content measured in the high-fat control group (Figure 2); no differences were noted among the pulses evaluated. The amount of *Akkermansia muciniphila* in the cecum relative to the high-fat control group was markedly elevated in bean- and lentil-fed mice but was unaffected in chickpea or dry pea-fed mice. Selective effects were also noted on bacteria in the phylum Firmicutes (no effect to suppression) and Bacteroidetes (increased 2.3- to 3.0-fold). When the fold change data were expressed as a ratio, the increase in Bacteroidetes to Firmicutes was increased 2.3 to 5.0 in pulse consuming mice, with the greatest increase in lentil-fed mice.

### 3.3. Effect of Pulses on Ileal FXR Expression

**Figure 3:**
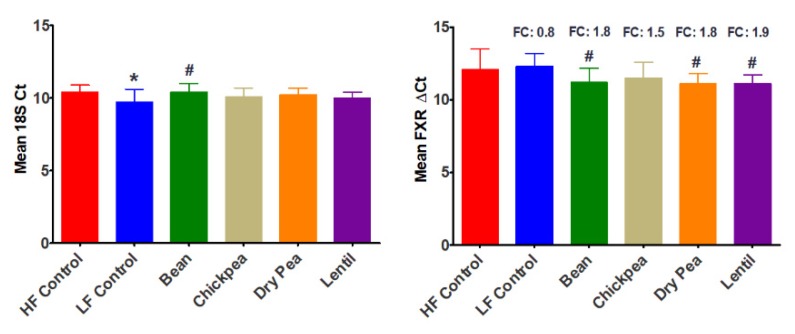
Farnesoid X Receptor (FXR) expression. (**A**) 18S Ct, ANOVA: LF Control *p* = 0.0268, Bean *p* = 0.9537, Chickpea *p* = 0.3215, Dry Pea *p* = 0.5767, Lentil *p* = 0.2114; (**B**) FXR ∆Ct, ANOVA: LF Control *p* = 0.6239, Bean *p* = 0.1225, Chickpea *p* = 0.2717, Dry Pea *p* = 0.1044, Lentil *p* = 0.0787; ^*^ different relative to HF Control (*p* ≤ 0.05); ^#^ different relative to LF Control (*p* ≤ 0.05). Fold change was calculated relative to HF Control; ANOVA: HF Control vs. all pulses *p* = 0.05, LF Control vs. all pulses *p* = 0.01, HF Control vs. LF Control *p* = 0.26; HF Control *n* = 7, LF Control *n* = 7, Bean *n* = 6, Chickpea *n* = 7, Dry Pea *n* = 8, Lentil *n* = 8; HF: high fat; LF: low fat.

Total RNA was isolated from the ileum and the level of FXR transcripts was determined by qPCR relative to 18S expression. Overall, pulse consumption increased FXR transcript levels relative to the high-fat control group by approximately 2-fold (*p* < 0.05); however, the differences among pulses were not statistically significant (Figure 3).

### 3.4. Effect of Pulses on Intestinal Morphometry

In order to further assess the effects of pulse consumption on intestinal function, morphometric, histochemical, and immunohistochemical techniques were applied to the ileum, and the ascending, transverse, and descending segments of the colon. The nature of these measurements as applied to the indicated intestinal segments is illustrated in Supplementary Figure S1. Only crypt height was affected by pulse consumption (Table 1). Pulses induced some changes in alcian blue staining and in the labeling index, but since these effects did not reach the level of statistical significance, the results are provided in Supplementary Table S4 and Table S5, respectively. Extension of crypt height was observed in pulse-fed mice in the transverse and descending segments of the colon. The magnitude of extension varied by pulse with the greatest extension being noted in lentil-fed mice. It is noteworthy that the longer crypts were not associated with either a change in mucin content of goblet cells (Supplementary Table S4) or a change in the magnitude of cell proliferation (Supplementary Table S5).

### 3.5. Effect of Pulses on Adiposity

Pulse consumption decreased both subcutaneous and visceral fat mass relative to the high-fat control, but fat mass was greater than that of the low-fat control group. Differences among pulses were not statistically significant. The reduction in fat mass was greater in the subcutaneous versus the visceral fat depot (Table 2).

### 3.6. Metabolite Variation among Pulse Types

**Figure 4:**
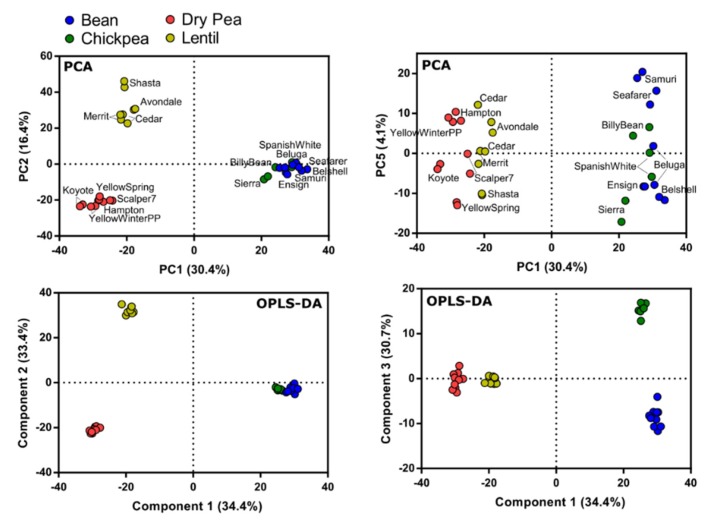
Metabolite variation among pulse types. Principal component analysis (PCA, top) and orthogonal projection to latent structures discriminant analysis (OPLS-DA, bottom) was performed on 17 varieties spanning four pulse types. The data matrix consisted of 2433 metabolites for *n* = 2 replicates per variety. Figures are scores plots for select components of the PCA and OPLS-DA.

Metabolite variation among pulse types was evaluated using a non-targeted mass spectrometry metabolomics approach. A total of 2433 metabolites were annotated and analyzed using principal component analysis (PCA). PCA characterized the lentil cultivar Pardina as an outlier and it was removed for all subsequent analysis. PCA with the outlier removed resulted in six components that explained 71% of the variation. The PCA demonstrated most of the variation as attributed to metabolite differences between pulse types (Figure 4). Specifically, PC1, PC2, and PC5 showed separation of the four pulse types, although bean and chickpea were highly similar. The PC5 separation of bean and chickpea (4.1%) was due to a lack of compounds in chickpea, and only one compound was identified to be higher in chickpea compared to the other pulses. Orthogonal projection to latent structures discriminant analysis (OPLS-DA) was performed to identify metabolites that were distinct to each pulse type, with bean, chickpea, dry pea, and lentil as the four classes within the model. The OPLS-DA resulted in three components that explained R^2^Y = 99% of the variation (Q^2^ = 89%). Analysis of the OPLS-DA loadings resulted in the characterization of 108 metabolites that were associated with differentiating the four pulse types (Supplementary Table S6 and Table S7). 

## 4. Discussion

Foods are considered dietary staples when they are eaten in large amounts throughout the day as a primary source of calories and protein. These foods are also affordable and accessible to the populations in which they serve as dietary staples. Grain legumes, also referred to as pulses, have been dietary staples since the dawn of agriculture [15]. However, in recent years a growing trend has been to replace them with animal derived foods and protein isolates [64]. Consequently, in many developed countries, pulses are now consumed infrequently by most of the population [2,65]. There are four pulse crops that are predominant in the world’s food supply: common bean, chickpea, dry pea, and lentil [15,66]. While most attention is given to pulses because they supply a large amount of protein in the absence of lipid, they are also an exceptional source of dietary fiber, containing 2–3 times more total fiber per 100 kcal edible portion than other foods commonly promoted as rich sources of dietary fiber [6,25,67]. 

Many barriers have obstructed progress in understanding how dietary fiber impacts human health. Prominent among these issues has been the lack of an internationally accepted consensus definition of dietary fiber and of an analytical approach that measures what is contained in that definition [68]. Consequently, the publication of a consensus definition and method in 2009 and 2011, respectively, was a landmark development in the field [69,70,71]. The definition and method divide dietary fiber components into three major categories: insoluble dietary fiber, soluble dietary fiber, and oligosaccharides. The variation among foods in these components as well as the investigation of human consumption in terms of these dietary fiber categories has been used to reflect differences in dietary fiber quality, but their association with health outcomes is controversial, and most recommendations continue to be framed in terms of total dietary fiber intake [6]. Nonetheless, given our recent work indicating that common bean, chickpea, dry pea, and lentil have similar levels of total dietary fiber [25], we decided that a comparative investigation of these pulse would provide an opportunity to compare the effects of similar amounts of dietary fiber from botanically similar but distinct sources. As discussed in the following sections, what is unknown is whether the bioactivity of pulses is equivalent. 

A number of recent studies have reported the effects of the consumption of various pulse crops on the fecal microbiome in preclinical models and numerous beneficial changes have been reported, although comparative studies among pulses have not been undertaken [19,22,24,72]. A major limitation of efforts to study the gut-associated microbiome in fecal specimens is that the majority of the commensal microbial species that have important effects are obligate anaerobes and the stability of these populations in feces is variable and appears to be short [73]. Recognizing this situation, and the report of differences between the cecal and fecal microbiome in mice fed chickpea [21], the bacterial analyses reported herein were made on the content of the cecum that was excised intact and immediately frozen in liquid nitrogen to preserve the anaerobic status of its content. The cecum is a segment of the gut that supports a diverse population of commensal microorganisms. Because the cecum sits at the intersection of the small and large intestine, its microbial content reflects the impact of dietary constituents that escape digestion in the small intestine. Consistent with expectations of a high fiber diet, rich in insoluble and soluble fermentable carbohydrates, the overall content of bacteria in the cecum was similarly increased by all pulses evaluated. Differential effects were also observed for the specific bacterium *Akkermansia munciniphila.* While lentil and common bean induced, respectively, a 49-fold and 25-fold increase in this bacterium relative to the high-fat control diet, chickpea and dry pea had no effect. The relevance of *A. muciniphila* is that colonization of the gut with this bacterium has been reported to be inversely associated with obesity, diabetes, and inflammation [74,75,76,77,78,79]. The differential effects of pulse consumption on the content of *A. muciniphila* is consistent with a selective effect limited to lentil and common bean. Given the emerging evidence of *A. muciniphila’s* health benefits, the identification of the beneficial prebiotic components of pulse crops is essential for at least two reasons. First, for lentil and common bean, in which the effect on *A. muciniphila* ranged between a 25- and 49-fold increase (Figure 2B), knowledge of prebiotic components could not only lead to the identification of cultivars within each crop with even stronger colony promoting activity, but could also prevent the loss of those compounds during food processing and the development of new food products. We also observed that pulses had differential effects on relative levels of bacteria in the phyla Bacteriodetes (increased) and Firmicutes (decreased). Given the controversial nature of the literature indicating whether an increase in the Bacteroidetes to Firmicutes ratio is consistent with health benefits [52,80,81,82,83,84], the importance of this observed is unclear. Nonetheless, the rank order of increase in comparison to the high-fat control diet was lentil (5-fold) > common bean (3.9-fold) > chickpea (2.9-fold) > dry pea (2.3-fold). For dry pea and chickpea, there are over 10,000 genetic variants of these pulses in various breeding programs and germplasm collections around the world [85]. Knowledge of the prebiotics that account for health-beneficial effects would provide an opportunity to guide development of cultivars of these crops that have more favorable gut health properties than those studied in this experiment. 

Since differences in microbial populations were observed when total dietary fiber levels were similar, it suggests an effect due to some aspect of dietary fiber quality. Because small molecules are known to be associated with dietary fiber, the small molecule profile of the pulses that were fed was evaluated and differences in composition were observed in classes of chemical compounds that have limited bioavailability in mammals and that microbes are known to metabolize [86,87]. For example, lentil was a distinct source of 3-fucosyllactose, which is an oligosaccharide identified in human milk and that has distinct beneficial effects on the human intestine. Of equal interest was the dominant presence of gulonic acid, a known substrate for bacteria in the *Bifidobacterium* genus. It is also likely that different carbohydrate moieties included within the framework of total dietary fiber vary among pulses in a manner that supports the propagation of different microbial colonies within the gut. 

There is a growing awareness of the value of increasing pulse consumption [1,64], in part because this will close the dietary fiber gap and decrease the risk for chronic diseases. However, as we recently reviewed [88], consumers have concerns about food tolerance that need to be addressed. The level of pulse consumption studied was intentionally high, i.e., approximately twice as high as median intakes of pulse consuming subgroups in the US and Canada. However, the dietary level was actually similar to dietary amounts consumed in developing countries where pulses are dietary staples. Moreover, the level incorporated into the diet (approximately 18g total dietary fiber/1000 kcal) would achieve levels of dietary intake in humans shown to reduce cardiometabolic disease related mortality by over 50% in the AARP cohort [14]. We examined several markers of intestinal function in either the ileum and/or the ascending, transverse, and descending segments of the colon. Crypt height, the rate of cell proliferation and its location within the crypt, and the amount of alcian blue positive mucin within goblet cells lining the intestinal lumen were assessed. The limited changes observed that were not statistically significant after adjustment for false discovery. This finding is consistent with our previous report that cooked pulses are well tolerated because anti-nutrients are completely inactivated by heat [89]. Our findings of normal gut physiology based on crypt size, mucus synthesis, and cell proliferation are consistent with the fact that pulse consumption at 2–3 times the intake observed in countries such as the United States and Canada is well tolerated. It is noteworthy that some laboratories have reported effects on the same intestinal morphometric parameters and have interpreted those effects as consistent with improved gut integrity. Our findings do not rebut those findings. Rather, our extensive experience in morphometric analyses recognized that differences in technical approaches can render different outcomes. In our judgement, the combined data of a number of publications [19,21,22,23,24,72] confirm that pulses are well tolerated and underscore the value of functional rather than morphometric assessment of gut integrity as an objective approach to moving the field forward.

Pulse consumption has been reported to improve blood lipid profiles associated with cardiovascular disease in clinical studies and in preclinical experiments in the model of obesity used in the work reported herein [42]. One possible contributor to pulse-mediated beneficial effects on circulating lipids involves changes in bile acid metabolism [89]. Since it has been shown that a shift in the ratio of Bacterioidetes to Firmicutes alters intestinal bile salt hydrolase activity in the ileum, and that such changes alter the expression of the bile acid receptor FXR [48,90,91,92], ileal transcript levels were assessed. Interestingly, all pulses induced expression of 1.5- to 1.8-fold relative to the obesogenic control diet. Whether this is enough to induce changes in bile acid metabolism in the liver associated with health benefits and/or results in changes in lipid metabolism in adipose tissue will require further detailed investigation. However, the fact that pulse consumption reduced lipid accumulation in subcutaneous and visceral adipose depots (Table 2) is consistent with mediation by alterations in bile acid metabolism and receptor driven signaling with FXR being a candidate transcription factor. 

This study has several limitations. They include (1) the inability to fully match chemical composition across dietary formulations when a whole food (pulse) approach is used; we argue that this approach is essential for understanding how the consumer may benefit from pulse crop consumption; (2) pulses were cooked and then immediately freeze dried; the potential impact of this approach on qualitative changes in carbohydrate constituents is unknown, but this approach is being used to make commercially available pulse powders that are being used as ingredients in the design of new food products; and (3) content of specific bacterial populations was determined by qPCR; while not as much information is obtained about overall microbial ecology as attained via high-throughput techniques, qPCR is a rapid, specific, and cost effect method [50].

## 5. Conclusions

Pulses are an affordable and accessible source of dietary protein and fiber. Encouraging the consumption of pulses as a dietary staple rather than as a vegetable side dish could eliminate the long-standing dietary fiber gap and result in levels of fiber intake that promote gut health and the prevention of four interrelated chronic diseases: obesity, type 2 diabetes, cardiovascular disease, and certain types of cancer in the absence of adverse outcomes. Our finding of differential effects of the four most commonly consumed, pulses, chickpea, common bean, dry pea and lentil, on specific health-beneficial commensal microbes residing in the cecum indicates the importance of defining the key prebiotic components of pulses so that producers can market value added cultivars of each pulse and consumers can be guided to the preparation and/or purchase of pulse-based foods that maintain health-beneficial effects. Studies of the impact of these pulses on the microbial ecology of each segment of the intestinal tract are also needed to fully understand their impact on intestinal function and to permit the assessment of whether fecal 16s rRNA profiling has merit as preclinical experiments are extended to clinical investigations.

## Figures and Tables

**Table 1:** Effect of feeding pulses on intestinal crypt height.

Diet ^1^	Ileum	Ascending Colon	Transverse Colon	Descending Colon
High-fat control	252.1 ± 41.1 ^a^	92.2 ± 9.3 ^b^	182.2 ± 23.7 ^f^	135.9 ± 9.6 ^k^
Low-fat control	262.4 ± 18.0 ^a^	93.3 ± 6.9 ^b, c^	207.3 ± 34.1 ^f, g^	150.1 ± 10.4 ^k, l^
Bean	274.5 ± 29.5 ^a^	82.2 ± 11.7 ^d^	214.1 ± 13.1 ^f, g, h^	153.9 ± 16.6 ^l, m^
Chickpea	269.7 ± 35.4 ^a^	89.5 ± 6.3 ^b, c, d^	239.1 ± 50.6 ^g, i^	139.6 ± 17.1 ^k, l, m^
Dry Pea	272.2 ± 27.5 ^a^	77.7 ± 7.6 ^d, e^	244.2 ± 53.2 ^g, i^	152.7 ± 14.6 ^l, m^
Lentil	255.6 ± 24.5 ^a^	86.9 ± 8.4 ^b, c, d^	257.6 ± 23.6 ^i, j^	166.0 ± 19.1 ^m, n^
***p*-values**				
	0.4676	0.0182	0.0015	0.0525

^1^ Values are crypt height (µm2) means ± SD; data compared using ANOVA and the Fisher least significant difference method for pairwise comparisons; different letter superscripts within a column indicate statistical significance *p* ≤ 0.5; Ileum ANOVA: HF Control vs. all pulses *p* = 0.22, LF Control vs. all pulses *p* = 0.69, HF Control vs. LF control *p* = 0.52; Ascending colon ANOVA: HF Control vs. all pulses *p* = 0.04, LF Control vs. all pulses *p* = 0.02, HF Control vs. LF control *p* = 0.82; Transverse colon ANOVA: HF Control vs. all pulses *p* < 0.01, LF Control vs. all pulses *p* = 0.04, HF Control vs. LF control *p* = 0.22; Descending colon ANOVA: HF Control vs. all pulses *p* = 0.02, LF Control vs. all pulses *p* = 0.63, HF Control vs. LF Control *p* = 0.12; Pulses include bean, chickpea, dry pea and lentil; High-fat control *n* = 7, Low-fat control *n* = 7, Bean *n* = 6, Chickpea *n* = 7, Dry Pea *n* = 8, Lentil *n* = 8; HF: high fat, LF: low fat.

**Table 2:** Effect of pulses on adiposity.

Diet ^1^	Subcutaneous Fat (mg/mm) ^2^	Sum Visceral Fat(mg/mm) ^2^	Tibia(mm)
High-fat control	138.7 ± 22.1 ^a^	246.5 ± 25.8 ^d^	17.8 ± 0.2 ^g, h, k, l^
Low-fat control	74.3 ± 13.8 ^b^	169.4 ± 14.7 ^e^	17.9 ± 0.2 ^h, k^
Bean	122.9 ± 15.8 ^c^	231.8 ± 8.2 ^d, f^	17.3 ± 0.5 ^i, j, k, l^
Chickpea	124.1 ± 22.1 ^c^	229.2 ± 22.6 ^f^	17.1 ± 0.3 ^j^
Dry Pea	119.2 ± 21.2 ^c^	226.6 ± 15.7 ^f^	17.6 ± 0.3 ^k, l^
Lentil	123.4 ± 20.8 ^c^	229.7 ± 22.2 ^d, f^	17.5 ± 0.3 ^l^
***p*-values**			
	< 0.0001	< 0.0001	0.0009

^1^ Values are means ± SD; ^2^ data compared using ANOVA with tibia length as a covariate and the Fisher least significant difference method for pairwise comparisons; different superscripts within a column indicate statistical significance *p* ≤ 0.5; ANOVA for subcutaneous fat: HF Control vs. all pulses *p* = 0.05, LF Control vs. all pulses *p* < 0.01, HF Control vs. LF Control *p* < 0.01; ANOVA for sum visceral fat: HF Control vs. all pulses *p* = 0.03, LF Control vs. all pulses *p* < 0.01, HF Control vs. LF Control *p* < 0.01; Pairwise comparisons: values within a column with different superscripts were statistically significant, p < 0.05. Pulses include bean, chickpea, dry pea and lentil; High-fat control *n* = 7, Low-fat control *n* = 7, Bean *n* = 6, Chickpea *n* = 7, Dry Pea *n* = 8, Lentil *n* = 8.

## Supplementary Materials

The following are available online at https://www.mdpi.com/2072-6643/12/3/593/s1. Figure S1: Intestinal tissue in high-fat control, Table S1: Composition of experimental diets, Table S2: Pulse food composition data, Table S3: qPCR Gene expression primers and bacterial primers, Table S4: Effect of feeding of pulses on intestinal alcian blue stained mucin, Table S5 Effect of feeding of pulses on intestinal cell proliferation, Table S6: Metabolites that discriminate pulse types, and Table S7: Metabolite data.
