# Supplementary material for: Pulse Crop Effects on Gut Microbial Populations, Intestinal Function, and Adiposity in a Mouse Model of Diet-Induced Obesity
Source: Nutrients. 2020 Feb 25;12(3):593. doi: 10.3390/nu12030593 (PMC7146478; doi:10.3390/nu12030593)
Supplement: Supplementary file 1 [file nutrients-12-00593-s001.zip › Supplementary Figure S1.docx]

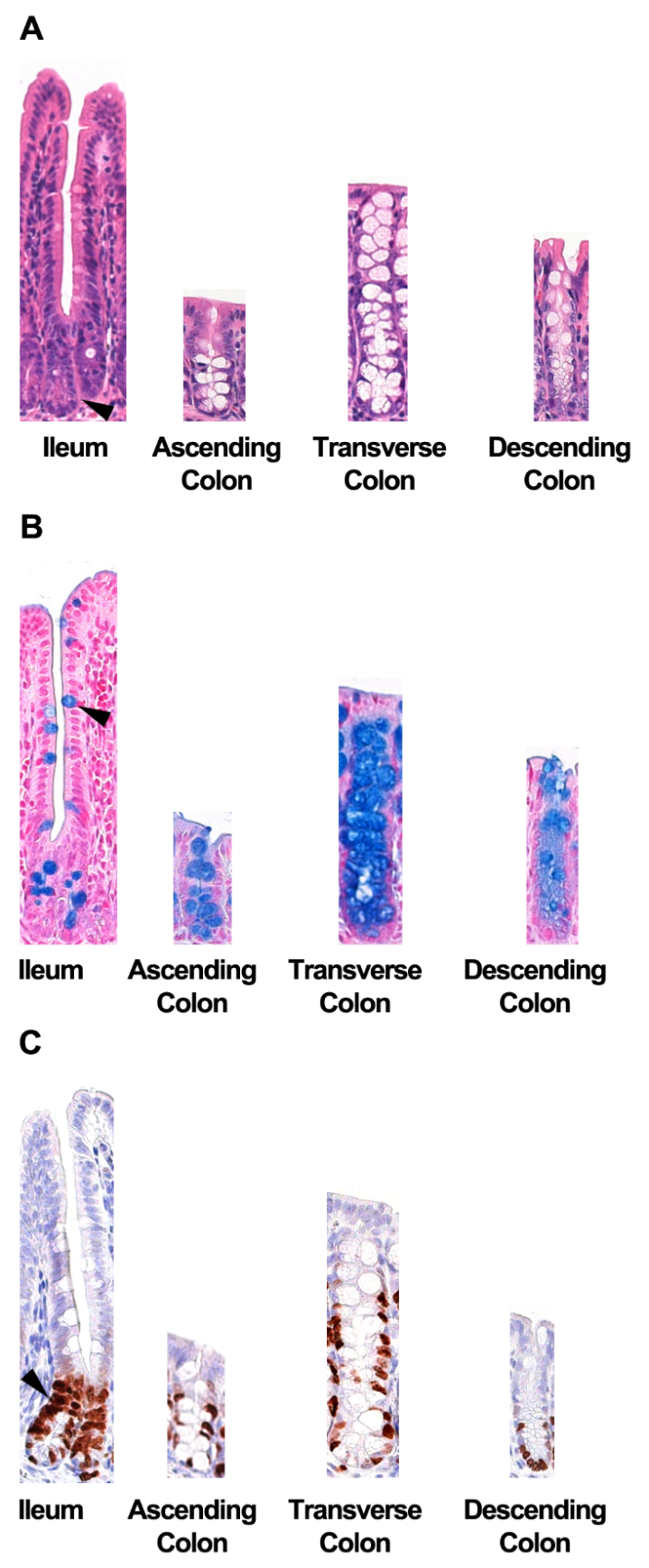


**Supplementary Figure S1.** Intestinal tissue from high fat control diet. (A) H&E stain showing difference in crypt height between ileum, ascending, transverse and descending segments of colonic tissue. Ileum contains bifurcated crypts (arrowhead); (B) Alcian blue nuclear fast red stain demonstrating goblet cells with alcian blue stained mucin. Fewer numbers of goblet cells are present in the ileum (arrowhead) compared with colonic segments and are much larger and more prominent in transverse colon; (C) Ki-67 positive nuclei (brown) present in the lower portion of the ileum (arrowhead) and colonic segments.
