# Supplementary material for: Pulse Crop Effects on Gut Microbial Populations, Intestinal Function, and Adiposity in a Mouse Model of Diet-Induced Obesity
Source: Nutrients. 2020 Feb 25;12(3):593. doi: 10.3390/nu12030593 (PMC7146478; doi:10.3390/nu12030593)
Supplement: Supplementary file 1 [file nutrients-12-00593-s001.zip › Supplementary Table S1.docx]

**Supplementary Table S1.** Composition of experimental diets.

| **Ingredient** | **Low Fat Control Diet ^1^ (g/100g)** | **High Fat Control Diet ^1, 2^**  **(g/100g)** | **Bean  Diet ^1, 2, 5^  (g/100g)** | **Chickpea Diet ^1, 2, 5^ (g/100g)** | **Dry Pea Diet ^1, 2, 5^ (g/100g)** | **Lentil Diet ^1, 2, 5^ (g/100g)** |
| --- | --- | --- | --- | --- | --- | --- |
| Solka-Floc | 4.7 | 6.5 | 0.0 | 0.0 | 0.0 | 0.0 |
| Cooked, Whole Pulse | 0.0 | 0.0 | 40.0 | 40.0 | 40.0 | 40.0 |
| Corn Starch | 29.9 | 0.0 | 0.0 | 0.0 | 0.0 | 0.0 |
| Casein (>=85% protein) | 19.0 | 25.8 | 17.1 | 17.1 | 17.1 | 17.1 |
| Cerelose (Dextrose) | 3.3 | 16.2 | 0.0 | 0.0 | 0.0 | 0.0 |
| Sucrose | 33.2 | 8.9 | 0.3 | 0.3 | 0.3 | 0.3 |
| Vitamin mix ^3^ | 1.0 | 1.3 | 1.3 | 1.3 | 1.3 | 1.3 |
| Cystine, L | 0 | 0 | 0 | 0 | 0 | 0 |
| DL-Methionine | 0.3 | 0.4 | 0.4 | 0.4 | 0.4 | 0.4 |
| L-Tryptophan (Sigma T0254-25G) | 0.00 | 0.00 | 0.01 | 0.01 | 0.01 | 0.01 |
| Choline bitartrate (41% choline) | 0.2 | 0.3 | 0.3 | 0.3 | 0.3 | 0.3 |
| Mineral mix ^4^ | 4.3 | 5.8 | 5.8 | 5.8 | 5.8 | 5.8 |
| Soybean oil | 2.4 | 3.2 | 3.2 | 3.2 | 3.2 | 3.2 |
| Lard | 1.9 | 31.7 | 31.7 | 31.7 | 31.7 | 31.7 |
| TOTAL (g) | 100.0 | 100.0 | 100.0 | 100.0 | 100.0 | 100.0 |

^1^ Experimental diets modified from the original diet formulations; ^2^ Dumas nitrogen(%) of complete diet mixture before oil was added: high fat, 6.3; chickpea, 5.8; dry pea, 6.3; lentil, 6.5; kidney bean, 7.0.; ^3^ Dyets #310025 AIN-93G vitamin mix. ^4^ Dyets #210025 AIN-93G mineral mix. ^5^ For each pulse treatment group the whole pulse was cooked and processed with the leachate, freeze dried and homogenized into a fine powder; varieties used in each pulse diet were as follows: Bean (White kidney); three varieties of Chickpea (Sierra, Spanish White, Billy bean); seven varieties of Dry Pea (Yellow Winter #124-7146, Koyote Yellow Winter, Yellow Spring 17 Pro # 093-7410, Yellow Spring Pro # 133-6243, Yellow Spring Pro#882, Hampton, Scalper 7); five varieties of Lentil (Merrit, Avondale, Cedar, Shasta, Pardina); pulse types containing 3 or more varieties were combined using equal portions of each variety.
