# Supplementary material for: Pulse Crop Effects on Gut Microbial Populations, Intestinal Function, and Adiposity in a Mouse Model of Diet-Induced Obesity
Source: Nutrients. 2020 Feb 25;12(3):593. doi: 10.3390/nu12030593 (PMC7146478; doi:10.3390/nu12030593)
Supplement: Supplementary file 1 [file nutrients-12-00593-s001.zip › Supplementary Table S2.docx]

**Supplementary Table S2.** Pulse food composition data.

| **Pulse ^1^** | **Protein  (g/100g)** | **Total dietary fiber  (g/100g)** | **Carbohydrate**  **(by difference) (g/100g)** |
| --- | --- | --- | --- |
| Chickpea | 8.86 | 7.6 | 21.1 |
| Dry Pea | 8.30 | 8.3 | 21.0 |
| Lentil | 9.02 | 7.9 | 19.5 |
| Kidney Bean | 8.62 | 7.4 | 21.2 |

^1^ Resource: USDA Food Data Central, <https://fdc.nal.usda.gov> ; cooked seed.
