# Supplementary material for: Pulse Crop Effects on Gut Microbial Populations, Intestinal Function, and Adiposity in a Mouse Model of Diet-Induced Obesity
Source: Nutrients. 2020 Feb 25;12(3):593. doi: 10.3390/nu12030593 (PMC7146478; doi:10.3390/nu12030593)
Supplement: Supplementary file 1 [file nutrients-12-00593-s001.zip › Supplementary Table S3.docx]

**Table 3.** qPCR Gene expression primers and bacterial primers.

| **Gene expression primers ^1^** | **Sequence** |
| --- | --- |
| 18S FWD | 5'- ATTGGAGCTGGAATTACCGC -3' |
| 18S REV | 5'- CGGCTACCACATCCAAGGAA -3' |
| FXR FWD | 5'- TGGGCTCCGAATCCTCTTAGA -3' |
| FXR REV | 5'- TGGTCCTCAAATAAGATCCTTGG -3' |
| SHP FWD | 5'- TCTGCAGGTCGTCCGACTATTC -3' |
| SHP REV | 5'- AGGCAGTGGCTGTGAGATGC -3' |
| FGF15 FWD | 5'- GCCATCAAGGACGTCAGCA -3' |
| FGF15 REV | 5'- CTTCCTCCGAGTAGCGAATCAG -3' |
| **Bacterial primers** | **Sequence** |
| 16S (926) FWD ^2^ | 5'- AAA CTC AAA KGA ATT GAC GG -3' |
| 16S (1062) REV ^2^ | 5'- CTC ACR RCA CGA GCT GAC -3' |
| *Akkermansia Muciniphila* FWD ^3^ | 5'- CAG CAC GTG AAG GTG GGG AC -3' |
| *Akkermansia Muciniphila* REV ^3^ | 5'- CCT TGC GGT TGG CTT CAG AT -3' |
| Bacteroidetes FWD ^4^ | 5'- AAA CTC AAA KGA ATT GAC GG -3' |
| Bacteroidetes REV ^4^ | 5'- GGT AAG GTT CCT CGC GCT AT -3' |
| Firmicutes (928) FWD ^4^ | 5'- TGA AAC TYA AGG AAT TGA CG -3' |
| Firmicutes (1040) REV ^4^ | 5'- ACC ATG CAC CAC CTG TC -3' |

^1^All gene expression primers previously reported in Jiang, C.; Xie, C.; Lv, Y.; Li, J.; Krausz, K.W.; Shi, J.; Brocker, C.N.; Desai, D.; Amin, S.G.; Bisson, W.H., et al. Intestine-selective farnesoid X receptor inhibition improves obesity-related metabolic dysfunction. Nat Commun **2015**, 6, 10166, doi:10.1038/ncomms10166; ^2^ Yang, Y.W.; Chen, M.K.; Yang, B.Y.; Huang, X.J.; Zhang, X.R.; He, L.Q.; Zhang, J.; Hua, Z.C. Use of 16S rRNA Gene-Targeted Group-Specific Primers for Real-Time PCR Analysis of Predominant Bacteria in Mouse Feces. Appl Environ Microb **2015**, 81, 6749-6756, doi:10.1128/Aem.01906-15. De Gregoris, T.B.; Aldred, N.; Clare, A.S.; Burgess, J.G. Improvement of phylum- and class-specific primers for real-time PCR quantification of bacterial taxa. J Microbiol Meth **2011**, 86, 351-356, doi:10.1016/j.mimet.2011.06.010; ^3^ Collado, M.C.; Derrien, M.; Isolauri, E.; de Vos, W.M.; Salminen, S. Intestinal integrity and Akkermansia muciniphila, a mucin-degrading member of the intestinal microbiota present in infants, adults, and the elderly. Appl Environ Microbiol **2007**, 73, 7767-7770, doi:10.1128/AEM.01477-07; ^4^ Koliada, A.; Syzenko, G.; Moseiko, V.; Budovska, L.; Puchkov, K.; Perederiy, V.; Gavalko, Y.; Dorofeyev, A.; Romanenko, M.; Tkach, S., et al. Association between body mass index and Firmicutes/Bacteroidetes ratio in an adult Ukrainian population. Bmc Microbiol **2017**, 17, doi:ARTN 120, 10.1186/s12866-017-1027-1.
