# Supplementary material for: Pulse Crop Effects on Gut Microbial Populations, Intestinal Function, and Adiposity in a Mouse Model of Diet-Induced Obesity
Source: Nutrients. 2020 Feb 25;12(3):593. doi: 10.3390/nu12030593 (PMC7146478; doi:10.3390/nu12030593)
Supplement: Supplementary file 1 [file nutrients-12-00593-s001.zip › Supplementary Table S4.docx]

**Supplementary Table S4.** Effect of feeding of pulses on intestinal alcian blue stained mucin.

| **Diet ^1^** | **Ileum** | **Ascending colon** | **Transverse colon** | **Descending colon** |
| --- | --- | --- | --- | --- |
| High Fat Control | 1170.5 ± 557.5 | 1335.5 ± 248.7 | 4344.1 ± 1274.9 | 1731.4 ± 360.6 |
| Low Fat Control | 1157.8 ± 310.6 | 1341.3 ± 246.6 | 4480.0 ± 1227.5 | 1726.5 ± 346.0 |
| Bean | 1105.3 ± 280.1 | 1215.1 ± 312.6 | 5711.3 ± 1518.2 | 1508.1 ± 263.1 |
| Chickpea | 1234.4 ± 289.1 | 1287.3 ± 330.7 | 4938.4 ± 828.3 | 1654.1 ± 365.0 |
| Dry Pea | 1216.7 ± 338.8 | 1074.3 ± 159.4 | 5262.7 ± 1377.5 | 1556.4 ± 263.3 |
| Lentil | 1131.5 ± 240.8 | 1266.7 ± 289.5 | 5098.7 ± 1251.7 | 2000.7 ± 756.0 |
| ***p*-values** | | | | |
|  | 0.9932 | 0.3344 | 0.1347 | 0.9700 |

^1^ Values are alcian blue stained area (µm^2^) means ± SD; Ileum ANOVA: HF Control vs all pulses *p* = 0.9781, LF Control vs all pulses *p* = 0.91, HF Control vs LF control *p* = 0.99; Ascending colon ANOVA: HF Control vs all pulses *p* = 0.26, LF Control vs all pulses *p* = 0.24, HF Control vs LF control *p* = 0.97; Transverse colon ANOVA: HF Control vs all pulses *p* = 0.10, LF Control vs all pulses *p* = 0.15, HF Control vs LF control *p* = 0.84; Descending colon ANOVA: HF Control vs all pulses *p* = 0.84, LF Control vs all pulses *p* = 0.86, HF Control vs LF control *p* = 0.98; Pulses include Bean, Chickpea, Dry Pea and Lentil; High Fat Control *n* = 7, Low Fat Control *n* = 7, Bean *n* = 6, Chickpea *n* = 7, Dry Pea *n* = 8, Lentil *n* = 8; HF: high fat, LF: low fat.
