# Supplementary material for: Pulse Crop Effects on Gut Microbial Populations, Intestinal Function, and Adiposity in a Mouse Model of Diet-Induced Obesity
Source: Nutrients. 2020 Feb 25;12(3):593. doi: 10.3390/nu12030593 (PMC7146478; doi:10.3390/nu12030593)
Supplement: Supplementary file 1 [file nutrients-12-00593-s001.zip › Supplementary Table S5.docx]

**Supplementary Table S5.** Effect of feeding of pulses on intestinal cell proliferation.

| **Diet ^1^** | **Ileum** | **Ascending colon** | **Transverse colon** | **Descending colon** |
| --- | --- | --- | --- | --- |
| High Fat Control | 26.3 ± 5.8 | 19.4 ± 3.4 | 19.1 ± 5.9 | 17.6 ± 2.0 |
| Low Fat Control | 24.9 ± 2.9 | 19.4 ± 3.9 | 22.5 ± 4.4 | 17.5 ± 3.7 |
| Bean | 26.4 ± 4.8 | 21.7 ± 3.2 | 22.9 ± 3.4 | 17.5 ± 1.3 |
| Chickpea | 21.9 ± 2.2 | 23.4 ± 2.9 | 26.6 ± 2.7 | 18.5 ± 2.5 |
| Dry Pea | 25.9 ± 3.9 | 21.0 ± 2.9 | 21.3 ± 3.1 | 18.6 ± 3.1 |
| Lentil | 23.7 ± 3.3 | 22.5 ± 3.0 | 23.8 ± 5.2 | 19.0 ± 4.4 |
| ***p*-values** | | | | |
|  | 0.5625 | 0.0425 | 0.1966 | 0.6527 |

^1^ Values are Ki-67 means ± SD, the percentage of Ki-67 positive cells were measured along the entire length of each crypt; Ileum ANOVA: HF Control vs all pulses *p* = 0.2874, LF Control vs all pulses *p* = 0.78, HF Control vs LF control *p* = 0.53; Ascending colon ANOVA: HF Control vs all pulses *p* = 0.05, LF Control vs all pulses *p* = 0.05, HF Control vs LF control *p* = 0.99; Transverse colon ANOVA: HF Control vs all pulses *p* = 0.08, LF Control vs all pulses *p* = 0.87, HF Control vs LF control *p* = 0.14; Descending colon ANOVA: HF Control vs all pulses *p* = 0.51, LF Control vs all pulses *p* = 0.45, HF Control vs LF control *p* = 0.93; Pulses include Bean, Chickpea, Dry Pea and Lentil; High Fat Control *n* = 7, Low Fat Control *n* = 7, Bean *n* = 6, Chickpea *n* = 7, Dry Pea *n* = 8, Lentil *n* = 8; HF: high fat, LF: low fat.
