# Supplementary material for: Pulse Crop Effects on Gut Microbial Populations, Intestinal Function, and Adiposity in a Mouse Model of Diet-Induced Obesity
Source: Nutrients. 2020 Feb 25;12(3):593. doi: 10.3390/nu12030593 (PMC7146478; doi:10.3390/nu12030593)
Supplement: Supplementary file 1 [file nutrients-12-00593-s001.zip › Supplementary Table S6.docx]

**Supplementary Table S6.** Metabolites That Discriminate Pulse Types.

| **Identifier^1^** | **Class** | **Metabolite^2^** | **Bean**  **(*z*)^3^** | **Chickpea  *(z)*** | **Dry Pea (*z*)** | **Lentil (*z*)** |
| --- | --- | --- | --- | --- | --- | --- |
| **BEAN** | | | | | | |
| GC_C070 | Amines | alanine | 1.01 | -0.18 | -1.25 | 0.44 |
| LCneg_C0144 |  | histidine | 1.12 | -0.07 | -0.86 | -0.27 |
| GC_C366 |  | leucine | 1.08 | 0.05 | -0.76 | -0.44 |
| GC_C068 |  | threonine | 0.97 | -0.09 | -1.19 | 0.35 |
| LCpos_C0969 |  | neamine^4^ | 1.20 | 0.00 | -0.76 | -0.55 |
| LCneg_C0747 | peptides | N2-gamma-glutamylglutamine | 1.11 | -0.11 | -0.54 | -0.63 |
| GC_C054 | purines | uridine | 1.04 | 0.02 | -0.86 | -0.23 |
| GC_C285 |  | xanthine | 0.98 | -0.40 | -0.58 | -0.20 |
| GC_C093 | saccharides | gulonic acid | 1.16 | -0.36 | -0.95 | 0.02 |
| GC_C467 |  | rhamnose | 1.00 | 0.11 | -0.77 | -0.37 |
| LCpos_C1130 | terpenes | araliasaponin III | 1.20 | -0.07 | -0.64 | -0.64 |
| LCpos_C1131 | (saponins) | soyasaponin A2 | 1.09 | -0.23 | -0.53 | -0.53 |
| LCneg_C1765 |  | camellioside A | 1.06 | 0.02 | -0.58 | -0.61 |
| LCpos_C0610 | terpenes (sterols) | 4alpha-carboxy-5alpha-cholesta-8-en-3beta-ol | 1.24 | -0.04 | -0.45 | -0.95 |
| LCpos_C0128 |  | tetrahydrodeoxycorticosterone | 1.09 | -0.45 | -0.46 | -0.45 |
| LCpos_C1198 | terpenes | methyl dihydrophaseate | 1.23 | -0.11 | -0.73 | -0.55 |
| LCneg_C1330 | (others) | all-trans-heptaprenyl diphosphate | 0.93 | -0.35 | -0.25 | -0.58 |
| LCpos_C1188 | others | Hv-NCC-1 | 1.21 | -0.34 | -0.59 | -0.53 |
| **CHICKPEA** | | | | | | |
| LCneg_C1114 | nucleotides | Uridine diphosphate-N-acetylglucosamine | -0.08 | 0.92 | -1.16 | 0.86 |
| **DRY PEA** | | | | | | |
| LCneg_C1374 | alkaloids | calystegine A3 | -0.74 | -0.77 | 1.26 | -0.08 |
| LCneg_C0089 | amines | gamma-glutamyltyrosine | -0.66 | -0.67 | 1.52 | -0.57 |
| LCneg_C0194 |  | glutamyltryptophan | -0.52 | -0.63 | 1.44 | -0.69 |
| LCneg_C0175 |  | n-phenylacetylaspartic acid | -0.69 | -0.70 | 1.50 | -0.48 |
| LCpos_C0993 |  | n-acetyl-l-glutamate 5-semialdehyde | -0.82 | -0.77 | 1.27 | 0.01 |
| GC_C058 |  | tryptophan | -0.17 | -0.75 | 1.39 | -0.96 |
| LCneg_C0588 | nucleosides | xanthosine | -0.75 | -0.86 | 1.37 | -0.13 |
| LCneg_C0897 | organic acids | Pimelic acid | -0.62 | -0.68 | 1.14 | -0.14 |
| LCneg_C1204 |  | Oxoglutaric acid | -0.72 | -0.65 | 1.26 | -0.20 |
| LCneg_C0007 | peptides | gamma-Glutamylphenylalanine | -0.62 | -0.63 | 1.50 | -0.64 |
| LCneg_C0565 | phenolics | 1-O-sinapoylglucose | -0.66 | -0.53 | 1.44 | -0.58 |
| LCpos_C0819 |  | 4-hydroxycinnamic acid | -0.64 | -0.63 | 1.51 | -0.62 |
| LCneg_C1412 |  | biochanin A | -0.61 | -0.60 | 1.42 | -0.57 |
| LCpos_C0092 |  | cinnamic acid | -0.61 | -0.64 | 1.52 | -0.66 |
| LCneg_C0196 |  | gentesic acid 5-O-glu | -0.64 | -0.64 | 1.48 | -0.58 |
| LCpos_C1180 |  | hydroxytyrosol 1-O-glu | -0.63 | -0.64 | 1.42 | -0.51 |
| LCneg_C1736 |  | kaempferol 3-(2G-apiosylrobinobioside) | -0.46 | -0.65 | 1.31 | -0.57 |
| LCpos_C0824 |  | sinapic acid | -0.48 | -0.59 | 1.34 | -0.63 |
| LCneg_C0585 | saccharides | a-L-fucopyranosyl-(1->2)-b-D-galactopyranosyl-(1->2)-D-xylose | -0.61 | -0.61 | 1.43 | -0.57 |
| LCpos_C0091 |  | cellobiose | -0.74 | -0.78 | 1.34 | -0.16 |
| LCneg_C1435 |  | stachyose | -0.60 | -0.66 | 1.43 | -0.54 |
| LCneg_C0068 |  | trisaccharide (raffinose-like) | -0.56 | -0.89 | 1.44 | -0.43 |
| LCpos_C0152 | terpenes | 22:0-Glc-Sitosterol | -0.50 | -0.53 | 1.50 | -0.85 |
| LCpos_C0918 | (sterols) | 22:0-Glc-Stigmasterol | -0.67 | -0.66 | 1.49 | -0.53 |
| LCpos_C0229 |  | (3beta,5alpha,6beta,9alpha,22E,24R)-23-Methylergosta-7,22-diene-3,5,6,9-tetrol | -0.35 | -1.13 | 1.19 | -0.19 |
| LCneg_C1366 | terpenes | (1S,2S,4R,8S)-p-Menthane-1,2,8,9-tetrol 2-glucoside | -0.66 | -0.65 | 1.49 | -0.56 |
| LCpos_C0887 |  | (E)-4,8-Dimethyl-1,3,7-nonatriene | -0.26 | -0.90 | 1.28 | -0.59 |
| LCneg_C1708 |  | dihydrophaseic acid | -0.52 | -0.53 | 1.27 | -0.54 |
| LCpos_C0603 | Others | 13'-Hydroxy-alpha-tocopherol | -0.76 | -0.76 | 1.51 | -0.36 |
| LCpos_C0182 |  | 1H-Indole-3-carboxaldehyde | -0.11 | -0.72 | 1.31 | -0.97 |
| LCpos_C0705 |  | 2-acetolactate | -0.58 | -0.58 | 1.38 | -0.57 |
| LCneg_C0617 |  | apiosylglucosyl 4-hydroxybenzoate | -0.71 | -0.72 | 1.30 | -0.20 |
| LCneg_C0894 |  | ethyl (S)-3-hydroxybutyrate glucoside | -0.56 | -0.57 | 1.30 | -0.50 |
| LCpos_C0218 |  | indole-3-acrylic acid | -0.21 | -0.70 | 1.39 | -0.94 |
| LCpos_C0544 |  | menaquinol-11 | -0.34 | -0.30 | 1.39 | -1.08 |
| LCpos_C0693 |  | pantothenic acid | -0.71 | -0.68 | 1.18 | -0.08 |
| LCpos_C0907 |  | plastoquinone 9 | -0.66 | -0.56 | 1.11 | -0.15 |
| LCpos_C0635 |  | pyropheophytin b | -0.46 | -0.44 | 1.15 | -0.53 |
| **LENTIL** | | | | | | |
| LCneg_C0082 | alkaloids | croomine | -0.44 | -0.44 | -0.37 | 1.34 |
| LCpos_C0107 |  | fauripavine | -0.42 | -0.40 | -0.56 | 1.52 |
| GC_C032 | amines | aminobutanoic acid | -0.05 | -1.32 | -0.30 | 1.43 |
| LCneg_C0288 |  | argininosuccinic acid | -0.32 | -0.81 | -0.48 | 1.60 |
| LCpos_C0298 |  | gamma-hydroxy-L-homoarginine | -0.48 | -0.53 | -0.53 | 1.66 |
| GC_C147 |  | glutamine, N-gamma-ethyl | -0.48 | -0.49 | -0.61 | 1.74 |
| LCneg_C0079 |  | homoarginine | -0.47 | -0.52 | -0.46 | 1.56 |
| LCneg_C0379 |  | hydroxyprolyl-leucine | -0.54 | -0.53 | -0.54 | 1.74 |
| LCpos_C0582 |  | kynurenic acid | -0.48 | -0.48 | -0.49 | 1.57 |
| LCneg_C1168 |  | N6-acetyl-l-lysine | -0.54 | -0.54 | -0.54 | 1.76 |
| LCpos_C0495 |  | N-acetylornithine | -0.17 | -0.37 | -0.70 | 1.37 |
| GC_C128 |  | threonine | -0.43 | -0.45 | -0.57 | 1.59 |
| GC_C148 |  | tryptamine | -0.48 | -0.48 | -0.46 | 1.53 |
| GC_C276 |  | ureidopropionic acid | -0.47 | -0.48 | -0.50 | 1.56 |
| LCneg_C1173 | organic acids | itaconic acid | -0.50 | -0.47 | -0.56 | 1.68 |
| LCneg_C1176 |  | oxoglutaric acid | -0.42 | -0.42 | -0.44 | 1.38 |
| LCneg_C1137 | phenolics | 2',7-Dihydroxy-4'-methoxy-8-prenylflavan 2',7-diglucoside | -0.47 | -0.48 | -0.40 | 1.45 |
| LCneg_C0879 |  | 4-hydroxycinnamic acid | -0.38 | -0.33 | -0.55 | 1.42 |
| LCneg_C1750 |  | apimaysin | -0.54 | -0.57 | -0.36 | 1.56 |
| GC_C171 |  | arbutin | -0.48 | -0.48 | -0.49 | 1.57 |
| LCneg_C0332 |  | aspalathin | -0.45 | -0.45 | -0.45 | 1.45 |
| GC_C514 |  | hydroquinone | -0.50 | -0.51 | -0.54 | 1.68 |
| LCpos_C1011 |  | kaempferol | -0.41 | -0.35 | -0.51 | 1.41 |
| LCpos_C0020 |  | kaempferol 3-[glucosyl-(1->3)-rhamnosyl-(1->2)-[rhamnosyl-(1->6)-galactoside]] | -0.53 | -0.53 | -0.54 | 1.74 |
| LCpos_C0833 |  | kaempferol 3-rhamnosyl-(1->2)-[glucosyl-(1->3)-(4'''-p-coumaroylrhamnosyl)-(1->6)-galactoside];Capilliposide II | -0.49 | -0.50 | -0.51 | 1.62 |
| LCneg_C0873 |  | lusitanicoside | -0.47 | -0.47 | -0.44 | 1.49 |
| LCpos_C0985 |  | manassantin A | -0.47 | -0.47 | -0.47 | 1.53 |
| LCpos_C0122 |  | nigrasin j | -0.37 | -0.38 | -0.54 | 1.42 |
| LCneg_C0087 |  | phlorin | -0.52 | -0.73 | -0.38 | 1.66 |
| LCpos_C1158 | purines | 5'-methylthioadenosine | -0.44 | -0.46 | -0.57 | 1.60 |
| LCneg_C1716 |  | 9-(beta-D-ribofuranosyl)zeatin | -0.53 | -0.51 | -0.35 | 1.49 |
| LCpos_C0184 |  | adenosine 5'-carboxamide | -0.48 | -0.48 | -0.49 | 1.59 |
| LCneg_C0612 | saccharides | 3-fucosyllactose^4^ | -0.51 | -0.56 | -0.53 | 1.72 |
| LCneg_C0008 |  | verbascose | -0.42 | -0.48 | -0.39 | 1.37 |
| LCneg_C0156 | terpene (iridoids) | asperuloside | -0.49 | -0.49 | -0.51 | 1.62 |
| LCneg_C1139 |  | cantleyoside^4^ | -0.54 | -0.54 | -0.51 | 1.72 |
| LCneg_C0058 |  | mollugoside^4^ | -0.53 | -0.53 | -0.53 | 1.73 |
| LCneg_C0326 |  | multiroside^4^ | -0.53 | -0.54 | -0.51 | 1.70 |
| LCneg_C0730 | terpenes (saponins) | araliasaponin IV | -0.49 | -0.50 | -0.51 | 1.63 |
| LCneg_C1400 |  | araliasaponin V | -0.38 | -0.36 | -0.62 | 1.52 |
| LCneg_C1757 |  | elatoside D | -0.49 | -0.59 | -0.32 | 1.47 |
| LCpos_C0558 | terpenes  (others) | beta-sitosterol glucoside-3'-O-hexacosanoicate | -0.64 | -0.54 | -0.41 | 1.71 |
| LCneg_C0083 |  | cucumegastigmane II | -0.45 | -0.45 | -0.48 | 1.49 |
| LCneg_C0065 |  | dihydrophaseic acid 4'-O-beta- glucopyranoside | -0.39 | -0.30 | -0.75 | 1.65 |
| LCneg_C1408 |  | L-linalool 3-[xylosyl-(1->6)-glucoside] | -0.40 | -0.46 | -0.44 | 1.40 |
| LCneg_C0874 |  | pisumionoside | -0.52 | -0.53 | -0.49 | 1.66 |
| LCneg_C0856 |  | uzarigenin 3-[xylosyl-(1->2)-rhamnoside] | -0.44 | -0.44 | -0.44 | 1.43 |
| LCneg_C0122 | others | 1,12-di-L-ascorbyl dodecanedioate | -0.38 | -0.45 | -0.51 | 1.46 |
| LCneg_C1379 |  | 1-Octen-3-yl primeveroside | -0.44 | -0.44 | -0.38 | 1.36 |
| LCneg_C0234 |  | chelidonic acid | -0.49 | -0.47 | -0.45 | 1.53 |
| LCneg_C0906 |  | linalool oxide D 3-[apiosyl-(1->6)-glucoside] | -0.49 | -0.47 | -0.45 | 1.53 |
| LCneg_C0917 |  | porphinehexacarboxylic acid | -0.49 | -0.49 | -0.44 | 1.53 |
| LCpos_C0818 |  | pyridoxine | -0.55 | -0.69 | -0.40 | 1.70 |

1: GC (gas chromatography); LC (liquid chromatography); pos (positive mode); neg (negative mode); Cxxxx (compound number). 2: Level 2 metabolite annotations based on spectral interpretation 3: *z* is for metabolite abundance comparisons of each legume type compared to the population of all legumes assayed 4: best match, but putative.
